# Effectiveness of biomarker-based exclusion of ventilator-acquired pneumonia to reduce antibiotic use (VAPrapid-2): study protocol for a randomised controlled trial

**DOI:** 10.1186/s13063-016-1442-x

**Published:** 2016-07-16

**Authors:** Thomas P. Hellyer, Niall H. Anderson, Jennie Parker, Paul Dark, Tina Van Den Broeck, Suveer Singh, Ronan McMullan, Ashley M. Agus, Lydia M. Emerson, Bronagh Blackwood, Savita Gossain, Tim S. Walsh, Gavin D. Perkins, Andrew Conway Morris, Daniel F. McAuley, A. John Simpson

**Affiliations:** Institute of Cellular Medicine, Newcastle University, Newcastle upon Tyne, UK; Centre for Population Health Sciences, University of Edinburgh, Medical School, Edinburgh, UK; Newcastle Clinical Trials Unit, Newcastle University, Newcastle upon Tyne, UK; Institute of Inflammation and Repair, University of Manchester, Manchester Academic Health Sciences Centre & Intensive Care Unit, Salford Royal NHS Foundation Trust, Greater Manchester, UK; Becton Dickinson Biosciences, Erembodegem, Aalst, Belgium; Intensive Care Unit, Chelsea and Westminster Hospital, Imperial College London, London, UK; Department of Medical Microbiology, Kelvin Building, The Royal Hospitals, Belfast, UK; Northern Ireland Clinical Trials Unit, Elliot Dynes Building, The Royal Hospitals, Belfast, UK; Centre for Experimental Medicine, Queen’s University Belfast, Belfast, UK; Public Health Laboratory, Heart of England NHS Foundation Trust, Birmingham, UK; MRC Centre for Inflammation Research, University of Edinburgh, Edinburgh, UK; University of Warwick and Heart of England NHS Foundation Trust, Coventry, UK; Division of Anaesthesia, Department of Medicine, University of Cambridge, Addenbrooke’s Hospital, Cambridge Biomedical Campus, Cambridge, UK; Regional Intensive Care Unit, Royal Victoria Hospital, Grosvenor Road, Belfast, UK

**Keywords:** Ventilator-acquired pneumonia, Biomarker, Antibiotic stewardship, Randomised controlled trial

## Abstract

**Background:**

Ventilator-acquired pneumonia (VAP) is a common reason for antimicrobial therapy in the intensive care unit (ICU). Biomarker-based diagnostics could improve antimicrobial stewardship through rapid exclusion of VAP. Bronchoalveloar lavage (BAL) fluid biomarkers have previously been shown to allow the exclusion of VAP with high confidence.

**Methods/Design:**

This is a prospective, multi-centre, randomised, controlled trial to determine whether a rapid biomarker-based exclusion of VAP results in fewer antibiotics and improved antimicrobial management. Patients with clinically suspected VAP undergo BAL, and VAP is confirmed by growth of a potential pathogen at > 10^4^ colony-forming units per millilitre (CFU/ml). Patients are randomised 1:1, to either a ‘biomarker-guided recommendation on antibiotics’ in which BAL fluid is tested for IL-1β and IL-8 in addition to routine microbiology testing, or to ‘routine use of antibiotics’ in which BAL undergoes routine microbiology testing only. Clinical teams are blinded to intervention until 6 hours after randomisation, when biomarker results are reported to the clinician. The primary outcome is a change in the frequency distribution of antibiotic-free days (AFD) in the 7 days following BAL. Secondary outcome measures include antibiotic use at 14 and 28 days; ventilator-free days; 28-day mortality and ICU mortality; sequential organ failure assessment (SOFA) at days 3, 7 and 14; duration of stay in critical care and the hospital; antibiotic-associated infections; and antibiotic-resistant pathogen cultures up to hospital discharge, death or 56 days. A healthcare-resource-utilisation analysis will be calculated from the duration of critical care and hospital stay. In addition, safety data will be collected with respect to performing BAL. A sample size of 210 will be required to detect a clinically significant shift in the distribution of AFD towards more patients having fewer antibiotics and therefore more AFD.

**Discussion:**

This trial will test whether a rapid biomarker-based exclusion of VAP results in rapid discontinuation of antibiotics and therefore improves antibiotic management in patients with suspected VAP.

**Trial registration:**

ISRCTN65937227. Registered on 22 August 2013. ClinicalTrials.gov, NCT01972425. Registered on 24 October 2013.

**Electronic supplementary material:**

The online version of this article (doi:10.1186/s13063-016-1442-x) contains supplementary material, which is available to authorized users.

## Background

Patients admitted to the intensive care unit (ICU) constitute a group vulnerable to healthcare-associated infection (HCAI), and consistent with that, antibiotic consumption in the ICU is considerably higher than in other hospital environments [1]. The growing global challenge of antimicrobial-resistance requires improved antibiotic stewardship. This judgment is, however, challenging in critically ill patients in whom clinical signs of infection are non-specific and where the consequences of missing a treatable infection may be significant.

Ventilator-acquired pneumonia (VAP) is a common ICU HCAI and highlights the challenges of antibiotic stewardship. Few clinical features have a specificity of greater than 60 % [2–4], and infection is confirmed in approximately 40 % of patients with suspected VAP [5]. The majority of patients with suspected VAP have antibiotics started at the point of clinical suspicion and de-escalation or discontinuation of antibiotics is possible after 2–3 days, when culture information becomes available. During this period, a significant proportion of patients will receive antibiotics that may not be indicated, and furthermore, if antibiotics are not discontinued in light of negative cultures, a full course of unnecessary antibiotics may be administered.

Addressing limitations in diagnostic methods for infections has the potential to improve antibiotic management by expediting the diagnostic process. Biomarkers of infection can act as rapid surrogate markers. In a recent multi-centre observational study [5], we validated the findings of a single-centre-derivation study [6, 7], showing that bronchoalveolar lavage (BAL) fluid biomarkers could form a reliable test to exclude VAP. Low concentrations of BAL fluid interleukin 1-beta (IL-1β) consistently have a strong negative predictive value (NPV) in both the derivation and validation cohorts. Furthermore, in the validation cohort, a combination of IL-1β and IL-8 could be used as a biomarker test to rule out VAP with high confidence, with a NPV of 1 and a post-test probability of 0 % (95 % confidence interval 0–9.2 %). These biomarkers are measured by cytometric bead array (CBA), which is a multi-plex, flow cytometric application that can be performed in approximately 6 hours, offering the potential for a rapid biomarker-based test in the ICU.

The aim of this randomised trial is to determine if, in adult patients with suspected VAP, the use of the additional rule-out biomarker test will improve antibiotic management and reduce antimicrobial use in comparison to decision making based on microbiology results alone.

## Methods/Design

**Fig. 1:**
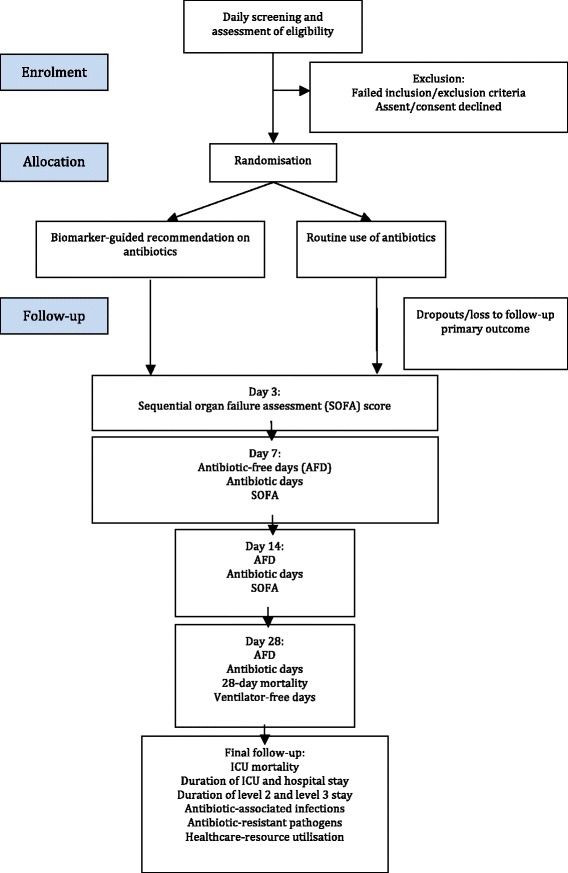
Consolidated Standards of Reporting Trials (CONSORT) 2010 flow diagram

This protocol outlines a multi-centre, prospective, controlled trial in which patients with suspected VAP are randomised 1:1 to a rapid biomarker-rule-out test in addition to standard care, compared to standard care alone (clinical judgment plus standard microbiological culture). The primary outcome measure is antibiotic-free days (AFD) in the 7 days following BAL. This clinical trial adheres to the Consolidated Standards of Reporting Trials statement (Fig. 1) [8] and principles of Good Clinical Practice.

### Study population

Participants are being recruited from the ICUs of 17 National Health Service (NHS) Trusts in the UK, for a total of 22 ICUs. These ICUs cover a broad case mix of medical, surgical and trauma patients, who are representative of current practice across the NHS.

Patients with suspected VAP who are at least 18 years of age, intubated and mechanically ventilated for 48 hours or more are considered eligible for inclusion in the trial. VAP is suspected based on the presence of a new or worsening chest X-ray or computed tomography (CT) changes consistent with pneumonia in the context of at least two of the following: temperature < 35 °C or > 38 °C; a white cell count of < 4 × 10^9^ / L or > 11 × 10^9^ / L; or purulent tracheal secretions [9]. Patients must also be considered suitable for early discontinuation of antibiotics by the clinical team (i.e. have no extra-pulmonary source of infection that mandates the use of continued antibiotics).

Patients are excluded on the basis of previously published criteria [10] that predict poor tolerance of the bronchoscopy and BAL that have been previously applied in our studies [5, 7]. These are: PaO_2_ < 8kPa on a FiO_2_ > 0.7; positive end-expiratory pressure of > 15 cm H_2_O; peak airway pressure > 35 cm H_2_O; heart rate > 140 beats per minute; mean arterial pressure < 65 mmHg; bleeding diathesis including platelet count < 20 × 10^9^/L or international normalised ratio (INR) > 3; poorly controlled intracranial pressure (>20 mmHg); or ICU consultant deems the procedure not to be safe. Patients may only be randomised to the study once (i.e. they are excluded if they have had previous BAL as part of the study). Patients are also excluded if assent/consent is declined.

Co-enrolment is allowed with observational studies. Co-enrolment with interventional studies is allowed following consideration of any scientific or statistical interactions in accordance with current UK recommendations [11].

### Biomarker assay and laboratory set up

The biomarker assay is performed in six NHS or university laboratories that act as testing ‘hubs’. Participating ICUs had to be within an expected travel time of 1.5 hours of a hub. BAL fluid samples are transported to the laboratory on ice immediately after sampling and measurement of BAL fluid IL-1β and IL-8 by cytometric bead array is performed with minimal delay on arrival. IL-1β and IL-8 are combined by logistic regression by the equation, established in the validation study [5]:

$$ -2.7385 + 1.4633 \times \log 10\left(1 + \mathrm{I}\mathrm{L}-1\upbeta \right)-0.2721 \times \log 10\left(1 + \mathrm{I}\mathrm{L}-8\right) $$

VAP is excluded if the regression output falls below a defined level (-1.7616).

All equipment, reagents and ongoing maintenance are supplied by the study’s industry partner, Becton Dickinson Biosciences (Franklin Lakes, NJ, USA). Each hub has been issued an Accuri C6 flow cytometer. This is a benchtop flow cytometer designed for its ease of use. The biomarker assay and all Accuri standard operating procedures (SOPs) have been designed such that they can be carried out by healthcare service laboratory technicians with limited flow cytometry experience. Centralised training in the biomarker assay and Accuri C6 was provided by investigators and scientists from Newcastle University and Becton Dickinson.

Accuri quality control tests are performed once a week by each hub, and these data are monitored by the flow cytometry core facility in Newcastle University. Technical support is provided by the investigators at Newcastle University and by Becton Dickinson.

### Intervention

All patients enrolled in the trial have suspected VAP and undergo the same clinical procedures of bronchoscopy and BAL. The BAL is performed according to a previously described SOP [5].

Patients are randomised to have BAL samples analysed by either the biomarker test in addition to semi-quantitative culture (the intervention arm) or semi-quantitative cultures alone (the control arm). The intervention arm is referred to as the ‘biomarker-guided recommendation on antibiotics’ group and the control arm the ‘routine use of antibiotics’ group. All semi-quantitative cultures are performed in a NHS or Public Health England laboratory and handled by a SOP in accordance with the UK Standards for Microbiological Investigation [12]. VAP is confirmed by the widely used threshold of growth of a potential pathogen at > 10^4^ colony-forming units per ml (CFU/ml) [13].

Biomarker results are reported to the clinical team by the technician using a standard script after approximately 6 hours. It is anticipated that all patients would have antibiotics started at the point of suspicion of VAP. In the event of a biomarker result that falls below the cut-off value, the clinical team is advised that VAP is excluded with high confidence and that early discontinuation of antibiotics is advised. If the biomarker value is above the threshold the clinical team is advised that VAP cannot be excluded and that standard care should continue.

### Risk to participants

Patients enrolled in this trial are by definition critically unwell, and minimising the risk to these patients is of paramount importance. BAL is an established and widely used technique for sampling the alveolar regions in ICU patients [13, 14]. Not only do eligibility criteria exclude patients who would poorly tolerate a BAL, but patients can be excluded based on the clinicians’ judgment of the risk profile.

The second consideration of risk is around the early discontinuation of antibiotics. The risk exists that antibiotics may be incorrectly discontinued in the face of undetected infection (i.e. in the setting of a false negative biomarker test). The validation study demonstrated that the threshold for excluding VAP using BAL IL-1β and IL-8 had an NPV of 1 which gives confidence in the test’s performance, but with a 95 % confidence interval of 0.92–1.0 false negatives remaining possible. Decisions around antibiotic prescribing are not dictated by the trial protocol, and these ultimately remain at the discretion of the treating clinician. This allows the clinician to restart antibiotics if it is felt they were discontinued inappropriately. This minimises the risk to patients and also makes the trial more pragmatic, testing its use in ‘real life’ clinical practice.

### Primary outcome and sample size

The primary outcome measure is the frequency distribution of AFD in the 7 days following BAL. This interval was used as this is the average reported duration of antibiotic therapy for suspected VAP in UK practice [15]. For the purposes of study design, AFD can be considered to be an integer value with patients in one of eight categories (0–7 AFD). Fewer days of antibiotic treatment will be detected as an increase in the proportion of patients with higher numbers of AFD and fewer patients having zero AFD. Antimicrobials delivered for prophylaxis will be excluded from analysis.

**Fig. 2:**
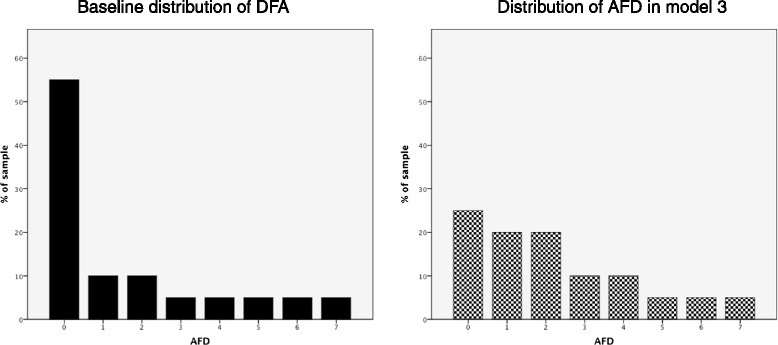
Graphical representation of the shift in distribution in antibiotic-free days (AFD) between baseline and model 3

The sample size is based on the frequency distribution of AFD in the 7 days after BAL in our previous validation study. This baseline distribution of AFD showed a skew towards patients with suspected VAP having few or no AFD (with this effect manifest both in those with ultimately confirmed VAP and those in whom BAL microbiology did not confirm VAP). We modelled changes in the distribution of AFD from baseline to a distribution with more patients having higher numbers of AFD. Table 1 gives examples of the different frequency distributions and their effect sizes. We judged a distribution that equates to effect sizes above 0.07 would represent a clinically important difference. By way of illustration, this would be equivalent to a change from a baseline median of 0 AFD (interquartile range 0–2.5 AFD) to a median of 1.5 AFD (interquartile range 0–3.5 AFD) under biomarker-guided treatment. An effect size of 0.0797 requires 90 patients per trial arm and allowing for a 15 % drop out, the total sample size is 210. This outcome measure and effect size was presented for national stakeholder peer review and judged to be appropriate (UK Critical Care Research Forum, July 2013). Figure 2 illustrates the changes in the AFD distribution between baseline and model 3 from Table 1.

**Table 1:** Models of different frequency distribution of AFD. Standard care distribution is based on data obtained from our validation cohort. The different models demonstrate increasing shifts in the frequency distribution towards more AFD in the sample. These distributions are illustrative, and different proportions in each category could give the same effect size. *AFD* antibiotic-free days

	Proportion of AFD (%)									
	0	1	2	3	4	5	6	7	N per arm	Effect size
Standard Care (from validation study)	55	10	10	5	5	5	5	5		
Model 1	40	20	15	5	5	5	5	5	215	0.033
Model 2	35	20	15	10	5	5	5	5	138	0.052
Model 3	30	20	15	10	10	5	5	5	96	0.075
Model 4	25	20	20	10	10	5	5	5	68	0.106

### Secondary outcome measures

Secondary outcome measures will include antibiotic days and AFD, expressed as continuous variables, at 7, 14 and 28 days; ventilator-free days at 28 days; 28-day mortality and ICU mortality; sequential organ failure assessment (SOFA) score at days 3, 7 and 14; duration of level 2 (high dependency unit) care, level 3 (intensive care unit) care and hospital stay; *Clostridium difficile* and MRSA infections up to hospital discharge, death or 56 days; and antibiotic-resistant pathogen cultures up to hospital discharge, death or 56 days. A healthcare-resource-utilisation analysis will be calculated from the duration of level 2 care, level 3 care and hospital stay up to discharge, death or 56 days.

Since this is a trial of a complex intervention, a process evaluation will be carried out in parallel with this trial. This will evaluate the process of conducting the trial and will aim to determine reasons for potential discrepancies in the expected and observed trial outcome, as well as providing information on the potential implementation of the intervention after trial completion. Additional files outline the detailed study protocol for the process evaluation (see Additional files 1 and 2).

### Data collection

Data are collected on the day of enrolment (day 0); day 3; day 7; day 14; day 28; and date of discharge, death or at 56 days. Clinical data collected include the age, gender, date of admission to the hospital and ICU, reason for admission to the hospital and ICU, functional comorbidities index, acute physiology and chronic health evaluation (APACHE) 2 score at admission, SOFA score, time from MV to suspected VAP, and pathogens cultured from BAL.

Safety measures will be recorded relative to the bronchoscopy and BAL. These will include SaO_2_, heart rate, blood pressure and PaO_2_:FiO_2_. Biological data will include concentrations of IL-1β and IL-8 in the BAL fluid for patients who are randomised to the intervention arm.

### Recruitment process and consent

All patients on the participating ICUs are screened on weekdays for eligibility. Potentially eligible patients are discussed with the ICU consultant to determine the appropriateness of early discontinuation of antibiotics and whether any other safety concerns are present that would exclude the patient.

Consent and assent procedures are in keeping with the legal framework of England, Northern Ireland (Mental Capacity Act, 2005) or Scotland (Adults with Incapacity (Scotland) Act, 2000) for consent/assent of adults without capacity. In England and Northern Ireland informed assent is obtained, where possible, following discussion with the patient’s next of kin (personal consultee). Where a personal consultee is unavailable, assent is provided by a nominated consultee, usually the ICU consultant, providing they are not also a member of the research team. In circumstances where the next of kin are unable to attend the ICU promptly, eliciting their opinion is possible by telephone to inform nominated assent.

In Scotland the patient’s relative or welfare attorney provides the informed consent. If the patient’s relative or welfare attorney is unable to attend the ICU, consent may be provided in a telephone conversation, providing a second member of staff witnesses the discussion.

Patients who recover capacity will be approached to provide retrospective informed consent. The decision as to whether that patient has regained capacity will reside with the treating team. The patient will be given sufficient time to consider the trial information before providing their consent to continued trial involvement.

### Randomisation and blinding

Once consent/assent is obtained, the clinical team informs the laboratory technician who will perform the biomarker test, and the technician then initiates randomisation through the Newcastle Clinical Trials Unit (NCTU). Randomisation is performed using a web-based randomisation service. Patients are randomised to the intervention arm or control arm in a 1:1 ratio by permuted blocks of variable length and stratified by site. The randomisation service generates either an instruction that the patient is randomised to ‘biomarker-guided recommendation on antibiotics: analyse sample on arrival’ or to ‘routine use of antibiotics: do not analyse sample on arrival’. This message is emailed to the technician.

The clinical team is initially blinded to the trial arm since all trial procedures are performed in all participants. Unblinding occurs when the biomarker result is called back to the clinical team after approximately 6 hours. To ensure consistency in unblinding, after this period, the clinical team are also informed if the patient was randomised to the control arm. The technician contacts the clinical team with the results according to local arrangements, which include contacting the on-call ICU consultant, ICU resident or the local principal investigator.

### Statistical analysis

Baseline clinical data will be compared between trial arms for balance using graphical and summary statistics appropriate for the data type of each variable. No formal tests of equality will be carried out. The primary outcome measure will be analysed by a chi-squared test on a 2 × 8 table of study arm versus AFD categories. Secondary outcome measures will be analysed by fitting appropriate generalised linear models with intervention and centre as covariates. Link functions will be determined by the type of outcome variable. A sub-group analysis for patients with trauma or head injury will be included as will a sub-group analysis based on clinician assessment of likelihood of VAP. Data analysis will be performed on an intention-to-treat basis, although other exploratory analyses including per-protocol analysis will be considered. A per-protocol analysis will be performed excluding patients who were randomised to the biomarker-guided recommendation on antibiotics arm, but who had a technical issue with the assay and therefore defaulted to standard care.

A within-trial cost analysis will be undertaken to assess the hospital resource use from the point of randomisation until hospital discharge or death, whichever occurs first, for a maximum of 56 days. Patient-level hospital resource use will be estimated from length of ICU stay and length of hospital stay. Multiple regression analyses will be performed to examine patient factors, which are potentially associated with costs. The robustness of the results will be evaluated using sensitivity analyses.

### Monitoring and adverse event reporting

A Data Monitoring and Ethics Committee will have oversight of the trial. This is an independent body that has oversight of safety data. They will make recommendations to the sponsor as to whether the trial should progress, be modified or terminated.

NCTU will monitor adherence to the trial protocol and completeness of the data collection. Adverse events (AE) and serious adverse events (SAE) that occur within 2 hours of BAL are reported to NCTU. Site investigators are able to report an AE/SAE outside of this period if they feel an AE/SAE is related to the study.

### Trial approvals, registration and status

Ethics approval for this trial has been granted by the National Research Ethics Service (England and Northern Ireland, Camberwell St Giles Committee, 13/LO/0651) and by the Scotland A Research Ethics Committee (13/SS/0074). Local research governance approval has been granted in the 17 NHS Trusts in which the trial is conducted: The Newcastle Upon Tyne NHS Foundation Trust; City Hospitals Sunderland NHS Foundation Trust; Heart of England NHS Foundation Trust; Salford Royal NHS Foundation Trust; Chelsea and Westminster NHS Foundation Trust; Lancashire Teaching Hospitals NHS Foundation Trust; Belfast Health and Social Care Trust; NHS Lothian; Countess of Chester NHS Foundation Trust; Northumbria Healthcare NHS Foundation Trust; University Hospitals Coventry and Warwickshire NHS Trust; Royal Liverpool and Broadgreen University Hospitals NHS Trust; Gateshead Health NHS Foundation Trust; Sandwell and West Birmingham Hospitals NHS Trusts; The Dudley Group of Hospitals NHS Foundation Trust; Central Manchester Hospitals NHS Foundation Trust; and South Tees Hospitals NHS Foundation Trust. The trial is registered on the ISRCTN registry (65937227) and on ClinicalTrials.gov (NCT01972425).

## Discussion

Limitations in diagnostic techniques to correctly rule-in or rule-out infections results in many patients receiving unnecessary antibiotics. Successfully validating the use of BAL fluid IL-1β and IL-8 to exclude VAP has been a significant step forward for developing diagnostics in this area [5]. A rapid rule-out of VAP should allow for discontinuation of antibiotics on the day of suspicion of VAP and therefore improve antibiotic management. This randomised controlled trial aims to determine the clinical utility of a rapid biomarker-based test by measuring the antibiotic use, which is expressed as AFD, in the 7 days that follow BAL.

Few biomarkers are used in the ICU to guide antibiotic management, and none specifically for VAP. Due to the novelty of the biomarker and the conduct of this trial in a complex clinical environment, regular education and reinforcement of the trial protocol is necessary with participating sites. Furthermore, the trial methodology accommodates the complexity of the intervention by including elements such as the process evaluation.

This trial represents a potentially important step forward for novel diagnostics in the ICU. If effective in its primary outcome it could result in an important improvement in antimicrobial stewardship in this patient group. It also represents a challenging and complex trial of novel diagnostics in a difficult clinical space. The process of conducting this trial will provide valuable information to inform future trials.

## Trial status

The trial is currently active in all participating sites. The first patient was recruited in December 2013, and the trial is due to complete in 2016.

## Abbreviations

AE, adverse event; AFD, antibiotic-free days; APACHE, acute physiology and chronic health evaluation; BAL, bronchoalveolar lavage; CBA, cytometric bead array; CFU, colony-forming units; CT, computed tomography; HCAI, healthcare-associated infection; ICU, intensive care unit; IL-1β, interleukin-1 beta; IL-8, interleukin-8; MRSA, methicillin-resistant *Staphylococcus aureus*; NCTU, Newcastle Clinical Trials Unit; NHS, National Health Service; NPV, negative predictive value; SAE, serious adverse event; SOFA, sequential organ failure assessment; SOP, standard operating procedure; VAP, ventilator-acquired pneumonia

## Additional files

Additional file 1: Process evaluation of the implementation and delivery of the VAPrapid-2 trial. Process evaluation protocol. (DOCX 33.9 kb) Additional file 2: VAPrapid-2 Logic Model. (PDF 87 kb)

## References

[1] Dumartin C, L’Hériteau F, Péfau M, Bertrand X, Jarno P, Boussat S (2010). Antibiotic use in 530 French hospitals: results from a surveillance network at hospital and ward levels in 2007. J Antimicrob Chemother.

[2] Torres A, El-Ebiary M, Padro L, Gonzalez J, de la Bellacasa JP, Ramirez J (1994). Validation of different techniques for the diagnosis of ventilator-associated pneumonia: comparison with immediate postmortem pulmonary biopsy. Am J Respir Crit Care Med.

[3] Fabregas N, Ewig S, Torres A, El-Ebiary M, Ramirez J, de La Bellacasa JP (1999). Clinical diagnosis of ventilator associated pneumonia revisited: comparative validation using immediate post-mortem lung biopsies. Thorax.

[4] Tejerina E, Esteban A, Fernandez-Segoviano P, Frutos-Vivar F, Aramburu J, Ballesteros D (2010). Accuracy of clinical definitions of ventilator-associated pneumonia: comparison with autopsy findings. J Crit Care.

[5] Hellyer TP, Morris AC, McAuley DF, Walsh TS, Anderson NH, Singh S (2015). Diagnostic accuracy of pulmonary host inflammatory mediators in the exclusion of ventilator-acquired pneumonia. Thorax.

[6] Wilkinson TS, Morris AC, Kefala K, O’Kane CM, Moore NR, Booth NA (2012). Ventilator-associated pneumonia is characterized by excessive release of neutrophil proteases in the lung. Chest.

[7] Conway Morris A, Kefala K, Wilkinson TS, Moncayo-Nieto OL, Dhaliwal K, Farrell L (2010). Diagnostic importance of pulmonary interleukin-1beta and interleukin-8 in ventilator-associated pneumonia. Thorax.

[8] Schulz KF, Altman DG, Moher D (2010). CONSORT 2010 statement: updated guidelines for reporting parallel group randomised trials. BMJ.

[9] American Thoracic Society (2005). Guidelines for the management of adults with hospital-acquired, ventilator-associated, and healthcare-associated pneumonia. Am J Respir Crit Care Med.

[10] Meduri GU, Chastre J (1992). The standardization of bronchoscopic techniques for ventilator-associated pneumonia. Chest.

[11] Krige A, Pattison N, Booth M, Walsh T, Walsh T, Fletcher S, et al. Co-Enrolment to Intensive Care Studies - A UK Perspective. J Intensive Care Soc. 2013;14(2):103–6.

[12] Health Protection Agency. UK standards for Microbiology investigations. Investigation of Bronchoalveolar Lavage, Sputum and Associated Specimens. Issued by the Standards Unit, Microbiology Services Division,Health Protection Agency, London. Issue date 02.08.12.

[13] Chastre J, Fagon JY (2002). Ventilator-associated pneumonia. Am J Respir Crit Care Med.

[14] Perkins GD, Chatterjee S, Giles S, McAuley DF, Quinton S, Thickett DR (2005). Safety and tolerability of nonbronchoscopic lavage in ARDS. Chest.

[15] Browne E, Hellyer TP, Baudouin SV, Conway Morris A, Linnett V, McAuley DF (2014). A national survey of the diagnosis and management of suspected ventilator-associated pneumonia. BMJ Open Respir Res.

