# Erratum to: ‘Effectiveness of biomarker-based exclusion of ventilator-acquired pneumonia to reduce antibiotic use (VAPrapid-2): study protocol for a randomised controlled trial’

**DOI:** 10.1186/s13063-016-1594-8

**Published:** 2016-09-26

**Authors:** Thomas P. Hellyer, Niall H. Anderson, Jennie Parker, Paul Dark, Tina Van Den Broeck, Suveer Singh, Ronan McMullan, Ashley M. Agus, Lydia M. Emerson, Bronagh Blackwood, Savita Gossain, Tim S. Walsh, Gavin D. Perkins, Andrew Conway Morris, Daniel F. McAuley, A. John Simpson

**Affiliations:** 1 Institute of Cellular Medicine, Newcastle University, Newcastle upon Tyne, UK; 2 Centre for Population Health Sciences, University of Edinburgh, Medical School, Edinburgh, UK; 3 Newcastle Clinical Trials Unit, Newcastle University, Newcastle upon Tyne, UK; 4 Institute of Inflammation and Repair, University of Manchester, Manchester Academic Health Sciences Centre & Intensive Care Unit, Salford Royal NHS Foundation Trust, Greater Manchester, UK; 5 Becton Dickinson Biosciences, Erembodegem Aalst, Belgium; 6 Intensive Care Unit, Chelsea and Westminster Hospital, Imperial College London, London, UK; 7 Department of Medical Microbiology, Kelvin Building, The Royal Hospitals, Belfast, UK; 8 Northern Ireland Clinical Trials Unit, Elliot Dynes Building, The Royal Hospitals, Belfast, UK; 9 Centre for Experimental Medicine, Queen’s University Belfast, Belfast, UK; 10 Public Health Laboratory, Heart of England NHS Foundation Trust, Birmingham, UK; 11 MRC Centre for Inflammation Research, University of Edinburgh, Edinburgh, UK; 12 University of Warwick and Heart of England NHS Foundation Trust, Coventry, UK; 13 Division of Anaesthesia, Department of Medicine, University of Cambridge, Addenbrooke’s Hospital, Cambridge Biomedical Campus, Cambridge, UK; 14 Regional Intensive Care Unit, Royal Victoria Hospital, Belfast, UK

## Erratum

Unfortunately, the original version of this article [1] contained an error. There are two references in the text to the threshold for defining VAP as greater than 10^4 colony forming units/ml. These should read greater OR EQUAL to 10^4 colony forming units/ml. The error is in the abstract and in the intervention sections.

In the abstract, please replace:

Patients with clinically suspected VAP undergo BAL and VAP is confirmed by growth of a potential pathogen at [>]10^4^ colony forming units per millilitre (CFU/ml).

With the following correct version:

Patients with clinically suspected VAP undergo BAL and VAP is confirmed by growth of a potential pathogen at [≥]10^4^ colony forming units per millilitre (CFU/ml).

In the Interventions, please replace:

VAP is confirmed by the widely used threshold of growth of a potential pathogen at [>]10^4^ colony forming units per ml (CFU/ml)(13).

With the following correct version:

VAP is confirmed by the widely used threshold of growth of a potential pathogen at [≥]10^4^ colony forming units per ml (CFU/ml)(13).
