# Supplementary material for: Effectiveness of biomarker-based exclusion of ventilator-acquired pneumonia to reduce antibiotic use (VAPrapid-2): study protocol for a randomised controlled trial
Source: Trials. 2016 Jul 16;17:318. doi: 10.1186/s13063-016-1442-x (PMC4947254; doi:10.1186/s13063-016-1442-x)
Supplement: Additional file 1: — Process evaluation of the implementation and delivery of the VAPrapid-2 trial. Process evaluation protocol. (DOCX 33.9 kb) [file 13063_2016_1442_MOESM1_ESM.docx]

**Process evaluation of the implementation and delivery of the VAPrapid-2 trial**

**Rationale**

Randomised trials are the most appropriate method for evaluating the comparative effectiveness of interventions [1], but when an intervention is complex it can be difficult to disentangle the effects of each of the interrelated and interdependent components and, as such, there are methodological challenges in determining the effects of these different components [2]. There is a need to address issues concerning how interventions work, the extent to which they are implemented as intended, and how patients who are, or are not, exposed to them react. A process evaluation of VAPrapid-2 will augment the trial by exploring all of the components of the intervention and providing greater explanatory power and understanding of the generalisability of the trial intervention and its implementation.

Developing a clear definition of the intervention and clarifying assumptions about how it works is fundamental to undertaking a process evaluation. Logic models are diagrammatic representations of how the intervention works, highlighting relationships between resources, activities, and intended outcomes. A logic model can uncover weaknesses in the assumptions, identify where clinicians have conflicting understandings of the intended intervention, or help evaluators to think critically about potential unintended consequences [3]. The VAPrapid-2 logic model defines: the current situation and the need for change; outputs in terms of what activities need to take place and who participates within them; and short, medium, and long-term outcomes [4] (Figure 1).

The process evaluation will examine three areas: fidelity, dose and reach.

1. The **fidelity** of intervention delivery is the extent to which the intervention is delivered as intended. This includes adherence to the components of the intervention and the trial (i.e. daily screening for VAP; undertaking a bronchoscopy and collecting BAL fluid and blood; randomisation; following the allocated intervention).
2. The intervention **dose** refers to how much of the intended intervention is delivered. In the context of this trial it examines to what extent changes were made in delivering the components and the decisions taken for operating ‘off protocol’.
3. The **reach** is the proportion of intended recipients who actually participate in an intervention. This includes the proportion of patients who were screened, excluded, recruited and completed the trial.

**Design**

The process evaluation will evaluate stages of the trial process including before, during, and after trial delivery using both quantitative and qualitative methods. Quantitative methods have the advantage of providing standardised data, but are limited in the degree to which they can illuminate more nuanced aspects of underlying processes through which outcomes are determined. Qualitative methods provide insights concerning *how* and *why* observed outcomes (anticipated and unanticipated, both positive and negative) are found. This process evaluation employs a mixed-methods design that draws upon data collected from screening logs routinely completed as part of the trial and semi-structured interviews with staff involved in the trial. The process evaluation draws upon data collection during the following phases:

1. *Pre-trial*: A service evaluation was conducted in each intensive care unit (ICU) to gather information on the ICU context (i.e. unit size and human resources) and differences in secular trends for VAP diagnosis and management across sites (e.g. the use of protocols; triggers for investigations; approach to the use of antibiotics and/or BAL). Data were collected from the site principal investigators by telephone or face-to-face interviews, using a structured data collection form to ensure standardisation of the information obtained. This information will be used to explain the impact of the change in practice of the new intervention on former practice.

2. *During the trial*: Quantitative and qualitative data recorded in the patient screening and recruitment logs will be utilised. The quantitative data includes the number of patients screened, excluded, included and randomised. These data will provide information to evaluate fidelity and reach of the intervention. Qualitative data from the screening and recruitment logs will include clinical decisions made during the trial. Research nurses will document reasons for patient exclusions (other than those outlined in exclusion criteria). In the situation that the patient has a biomarker result that excludes VAP but the ICU consultant does not follow the recommendation, the ICU consultant will be asked to complete a short questionnaire to provide information on clinical reasoning behind the decision. This will provide data to explain fidelity, dose and reach. Research nurses will be contacted regularly in order to ensure that data needed for the process evaluation are being recorded and contain sufficient detail.

3. *After the trial*: Semi-structured interviews (individual or focus group) will be undertaken with doctors, nurses, ward managers, and laboratory technicians involved in trial implementation or intervention delivery. Approximately 8-10 sites will be selected for these interviews following analysis of the pre and during trial data with respect to factors that facilitated or hindered implementation on sites. A matrix-sampling frame will be used to aim for maximum variation in selection of sites to enable a balance of sites where implementation was high, moderate or low and explore fidelity, dose, and reach. Clinician experiences and understandings of the VAPrapid-2 intervention trial will be explored in the context of the site, including those relating to barriers and facilitators to the delivery of the intervention. Areas that will be probed are time/resources, environmental practicalities, and staff attitudes/perceptions. The interview schedule will be determined by information collated from contextual and during-trial data collected throughout the VAPrapid-2 trial. Purposive sampling will be used to ensure a range of participants (e.g. according to grade, profession) to discuss their experiences of trial conduct and intervention delivery, including ICU culture, environment of care as well as wider organisational setting. The interviews will take place before the results of the trial are known, in order to avoid this knowledge from influencing views about the trial or the intervention. All interviews will be digitally recorded, audio files will be stored on a password-protected drive and audio files on the Dictaphone will be erased. Audio files will be transcribed in full, removing any identifiable information and saved in Microsoft Word (Microsoft Corporation, Redmond WA, USA) format. The transcripts will be stored on a password-protected computer and each participant will have a unique identification number.

**Data analysis**

*Process data analysis*

Descriptive statistics will summarise numerical data from screening and recruitment logs. A Framework approach will be used to analyse interview data that allows simultaneous analysis across themes and cases [5]. This approach is a matrix-based method for analysing data that includes familiarisation with the data, indexing the data and creating summaries from the indexed data. An important feature of this approach is that it allows themes or concepts identified *a priori* to be specified as indexing categories from the outset and to be combined with other themes or concepts that emerge *de novo* by subjecting the data to inductive analysis. To ensure confirmability and trustworthiness, a sample of transcripts will be double coded and the research team will review the thematic framework as it is applied to the data. Finally, the themes will be mapped and interpretation of those themes will be reviewed, to construct overall explanations of the data. A practical benefit of doing this is that it enables questions or issues identified both before and after knowledge of trial outcomes to be explicitly and systematically considered in the analysis, while also facilitating sufficient flexibility to detect and characterise issues that emerge from the data.

*Integrating process and outcome data*

The pre- and post-trial qualitative evidence will be systematically combined with the outcome data to identify the processes mediating the nature of trial implementation, receipt and setting and observed outcomes. The extent to which the intervention was implemented and the extent to which on-treatment analyses (on basis of receiving the intervention) might yield different results, will be explored. Process data will be used to generate hypotheses concerning factors and the mechanisms of their interaction likely to impact on outcomes. These hypotheses will be tested against the outcome data using statistical analyses to provide answers to research questions.

An example hypothesis may be ‘biomarker-guided recommendation on antibiotics is more effective in ICUs in which the intervention was most fully implemented and for patients who received the intervention than for those who did not’. To explore this, process evaluation data will be combined with outcome data in on-treatment analyses and compared with outcome data in intention-to-treat analysis. Following Oakley et al. [6], these are likely to include analyses in which:

- outcomes for patients who are randomly allocated to the intervention (on-treatment) are compared with results for patients who receive standard care;
- outcomes for patients in the ICUs where fidelity is high compared with patients in ICUs where the fidelity is low;
- outcome data in on-treatment analyses will be compared with outcome data in the intention-to-treat analysis.

Further hypotheses will be tested, including analyses to examine the relationship between key dimensions of the intervention, subgroups of ICUs and patients most and least likely to achieve positive outcomes from the intervention, and trial outcomes. In line with recommended practice, relevant theory will be drawn upon to help understand the observed relationships between process evaluation and trial outcome data [7].

**Acknowledgements**

This work is supported by the Medical Research Council (MRC) for a MRC Network of Hubs for Trials Methodology Research Fellowship (LME); and the Northern Ireland Public Health Agency, Research & Development Office.

**References**

[1] Gossop M, Marsden J. Assessing methodological quality of published papers. Pre-allocation bias in randomised controlled trials must be taken into account. British Medical Journal. 1998; 316(7125):151.

[2] Blackwood B. Methodological issues in evaluating complex healthcare interventions. Journal of Advanced Nursing. 2006; 54(5):612-622.

[3] Moore GF, Audrey S, Barker M, Bond L, Bonell C, Hardeman W et al. Process evaluation of complex interventions: Medical Research Council guidance. 2015; 350:h1258

[4] Taylor-Powell E, Steele S, & Douglah, M. Planning a program evaluation. http://www.uwex.edu/ces/pdande/evaluation/evallogicmodel.html (1996). Accessed 15 June 2015.

[5] Ritchie J, Spencer L. Qualitative data analysis for applied policy research. In: Bryman A, Burgess RG, editors. Analysing Qualitative Data. London: Routledge; 1994. p. 173-94.

[6] Oakley A, Strange V, Bonell C, Allen E, Stephenson J, & RIPPLE Study Team. Process evaluation in randomised controlled trials of complex interventions. British Medical Journal. 2006; 332(7538):413-416.

[7] May C, Finch T. Implementing, embedding, and integrating practices: An outline of Normalization Process Theory. Sociology. 2009; 43(3):535-554.

**Figure 1: Logic model for VAPrapid-2 trial.**
