# Supplementary material for: Effectiveness of biomarker-based exclusion of ventilator-acquired pneumonia to reduce antibiotic use (VAPrapid-2): study protocol for a randomised controlled trial
Source: Trials. 2016 Jul 16;17:318. doi: 10.1186/s13063-016-1442-x (PMC4947254; doi:10.1186/s13063-016-1442-x)
Supplement: Additional file 2: — VAPrapid-2 Logic Model. (PDF 87 kb) [file 13063_2016_1442_MOESM2_ESM.pdf]

VAPrapid-2 Logic Model

Situation: Suspected VAP is only confirmed in approximately 30% of cases - there is a need to improve antibiotic stewardship.

Inputs	Outputs – Activities	Outputs – Participation	Outcomes -- Impact – Short	Outcomes -- Impact – Medium	Outcomes -- Impact – Long
**Staff:**<br><br>Research nurse<br><br>Staff nurse<br><br>Clinician<br><br>Laboratory technician<br><br>**Resources:**<br><br>Bronchoscopy equipment<br><br>Staff time<br><br>Laboratory availability<br><br>Funding	Screen patient<br><br>Recruit patient<br><br>Select appropriately trained staff<br><br>Undertake sampling process<br><br>Sample processed at laboratory<br><br>Return results in a timely fashion<br><br>Willingness to participate<br><br>Belief in the intervention	Patient<br><br>Family member<br><br>Research nurse<br><br>Staff nurse<br><br>Clinician<br><br>Principal investigator and/or co-investigator<br><br>Laboratory technician	Test result<br><br>Antibiotics discontinued<br><br>Antibiotics continued<br><br>Development of trial skill competencies<br><br>Others trained in trial skills<br><br>Increase in knowledge in ICU of the management/diagnosis of VAP	Improved antibiotic stewardship<br><br>Introduction to the concept of using biomarkers as a tool to improve antibiotic stewardship<br><br>Improved diagnosis of VAP<br><br>Increase in knowledge in ICU of the management/diagnosis of VAP	Decrease AMR in the wider population<br><br>Sustained antibiotic stewardship<br><br>Reduced healthcare resource usage<br><br>Increase in knowledge in ICU of the management/ diagnosis of VAP

Assumptions

Sites will appropriately identify suspected VAP.
The prevalence of VAP will be same as the validation study (approx. 30%).
Patients will be recruited into the VAPrapid-2 trial.
Patient’s antibiotic therapy will follow trial protocol.
The VAPrapid-2 standard operating procedure is clinically acceptable.
Clinical staff see the test as beneficial at both individual and societal level, and engage with it.

External Factors

Normal working practices e.g. overnight antibiotics started by a trainee unfamiliar with the trial.
Balancing other work commitments - where the trial lies in priorities.
Decisions made outside of the trial protocol.
Role of microbiology in prescribing decisions.
Individual perception of risk-taking.
Attitude towards / perception of undertaking a bronchoscopy, and that of the biomarker test.
Other sources of infection.
